# Multi-Domain Convolutional Neural Networks for Lower-Limb Motor Imagery Using Dry vs. Wet Electrodes

**DOI:** 10.3390/s21196672

**Published:** 2021-10-07

**Authors:** Ji-Hyeok Jeong, Jun-Hyuk Choi, Keun-Tae Kim, Song-Joo Lee, Dong-Joo Kim, Hyung-Min Kim

**Affiliations:** 1 Biomedical Research Division, Bionics Research Center, Korea Institute of Science and Technology, Seoul 02792, Korea; jjh4599@korea.ac.kr (J.-H.J.); sodangdog@kakao.com (J.-H.C.); ktkim@kist.re.kr (K.-T.K.); songjoolee@kist.re.kr (S.-J.L.); 2 Department of Brain and Cognitive Engineering, Korea University, Seoul 02841, Korea; 3 Division of Bio-Medical Science & Technology, KIST School, Korea University of Science and Technology, Seoul 02792, Korea; 4 Department of Neurology, Korea University College of Medicine, Seoul 02841, Korea; 5 Department of Artificial Intelligence, Korea University, Seoul 02841, Korea

**Keywords:** brain–computer interfaces, electroencephalography, motor imagery, lower limb, electrodes, neural networks, multilayer neural network

## Abstract

Motor imagery (MI) brain–computer interfaces (BCIs) have been used for a wide variety of applications due to their intuitive matching between the user’s intentions and the performance of tasks. Applying dry electroencephalography (EEG) electrodes to MI BCI applications can resolve many constraints and achieve practicality. In this study, we propose a multi-domain convolutional neural networks (MD-CNN) model that learns subject-specific and electrode-dependent EEG features using a multi-domain structure to improve the classification accuracy of dry electrode MI BCIs. The proposed MD-CNN model is composed of learning layers for three domain representations (time, spatial, and phase). We first evaluated the proposed MD-CNN model using a public dataset to confirm 78.96% classification accuracy for multi-class classification (chance level accuracy: 30%). After that, 10 healthy subjects participated and performed three classes of MI tasks related to lower-limb movement (gait, sitting down, and resting) over two sessions (dry and wet electrodes). Consequently, the proposed MD-CNN model achieved the highest classification accuracy (dry: 58.44%; wet: 58.66%; chance level accuracy: 43.33%) with a three-class classifier and the lowest difference in accuracy between the two electrode types (0.22%, d = 0.0292) compared with the conventional classifiers (FBCSP, EEGNet, ShallowConvNet, and DeepConvNet) that used only a single domain. We expect that the proposed MD-CNN model could be applied for developing robust MI BCI systems with dry electrodes.

## 1. Introduction

A brain–computer interface (BCI) is a system that decodes the user’s intent from brain signals and allows the user to control a computer or other external device without actual movement [1,2,3]. BCIs are divided into invasive and noninvasive types depending on whether the decoding device for collecting brain signals requires surgical installation. Among the noninvasive BCIs that do not necessitate surgery, electroencephalography (EEG)-based BCI [4] is noted for its high temporal resolution [5], portability [6], and inexpensiveness [7,8]. BCI researchers have developed various paradigms that utilize signals such as evoked potentials (EPs) [9,10], steady-state visual evoked potentials (SSVEPs) [11,12], steady-state somatosensory evoked potentials (SSSEPs) [13,14], and motor imagery (MI) [15,16,17] to analyze and classify the intentions of BCI users.

EEG-based BCIs can be divided into dry electrode-based BCIs (dry electrode BCIs) [18] and wet electrode-based BCIs (wet electrode BCIs) [19], depending on the type of electrode used to obtain the EEG signal. Wet electrode BCI has been widely used for a variety of research purposes but has practical limitations involving discomfort with the wet gel, time constraints, and wearing time [20,21,22,23], but it achieves low impedance and high signal-to-noise ratio (SNR) signals due to the conductive gel placed between the electrode and the skin [21,24]. Dry electrode BCI, which measures EEG signals through spike electrodes that directly touch the scalp without the use of wet gels, has practical aspects that solve the constraints of wet gels but produces low SNR and high impedance signals [25,26,27]. Despite these limitations, the practicality of using dry electrode BCI is an attractive advantage that cannot be abandoned.

Therefore, several studies have attempted to solve these low signal quality issues and thus improve the performance of dry electrode BCIs [20,23,28,29]. Hua et al. developed semi-dry electrodes to compensate for the problems of wet electrodes, and obtained high-quality EEG signals from hair-covered electrode placement sites and presented low impedance signals and temporal correlations with wet electrodes to show the performance of their system [28]. Di Flumeri et al. also conducted comparative studies on the signal spectra and mental state classification aspects related to three different dry electrodes by measuring EEG signals and showed that the different dry electrode equipment could be conveniently placed and had comparable EEG signal results [20]. However, these hardware-based comprehensive comparison approaches [20,28,29,30] have focused on demonstrating high signal quality or a high SNR for new electrodes, mostly by demonstrating how similar the measured EEG data are to those obtained with wet electrodes. Furthermore, dry electrode development investigations and comprehensive comparative studies between dry electrode and wet electrode BCIs have been conducted using EP [27,31,32], SSSEP [33], and SSVEP [34], demonstrating the applicability of dry electrode BCI systems. However, only a few studies have compared dry and wet electrode BCIs with MI-based paradigms, either by limited use of MI classification algorithms [23] or by different subject conditions, in which only two out of six subjects performed MI with the wet electrode [25]. Therefore, more research is still needed on the effectiveness and performance improvements of dry electrode BCI in MI-based applications.

Recent BCI studies have shown the potential of using convolutional neural network (CNN)-based classifiers to improve dry electrode BCI. Schirrmeister et al. [35] demonstrated that ConvNet was robust to noise by not misclassifying experimental trials even when random frequency or amplitude noise was introduced. Kojoma et al. [26] also proposed a method to decode wet electrode signals from dry electrode signals with restoration filters learned from simultaneously measured dry and wet electrode signals. However, few studies of these state-of-the-art CNN-based MI BCI classifier algorithms have yet directly confirmed and compared the classification performance between dry and wet electrodes with the same number of channels and identical subject conditions.

The main contributions of our study are that it compared the performance achieved with dry and wet electrode BCI, and proposes a novel multi-domain CNN (MD-CNN) model which can reduce the performance gap between dry and wet electrode MI BCIs by improving the classification performance of dry electrode BCI. The MD-CNN’s multilayer structure can extract and learn suitable MI-related EEG features from multiple input data [36,37,38], and its multi-domain input can contain multiple EEG features that are less affected by the dry electrode’s low SNR or subject-specific differences [39]. The MD-CNN model combines the multilayer structure and the multi-domain input to improve the classification accuracy of dry electrode MI BCIs by learning multi-domain inputs with multiple layers to extract and classify EEG features from low SNR EEG signals. We first investigated the classification accuracy on the BCI Competition IV dataset 2a [40] to evaluate the performance of our proposed MD-CNN as an MI BCI classifier. We then validated the MI BCI classification accuracy of the proposed MD-CNN by recording the signals obtained with dry and wet electrode BCI systems from 10 subjects over two sessions. We also compared the classification accuracy among four different MI BCI classifiers.

## 2. Materials and Methods

### 2.1. Multi-Domain CNN Model Architecture

**Figure 1:**
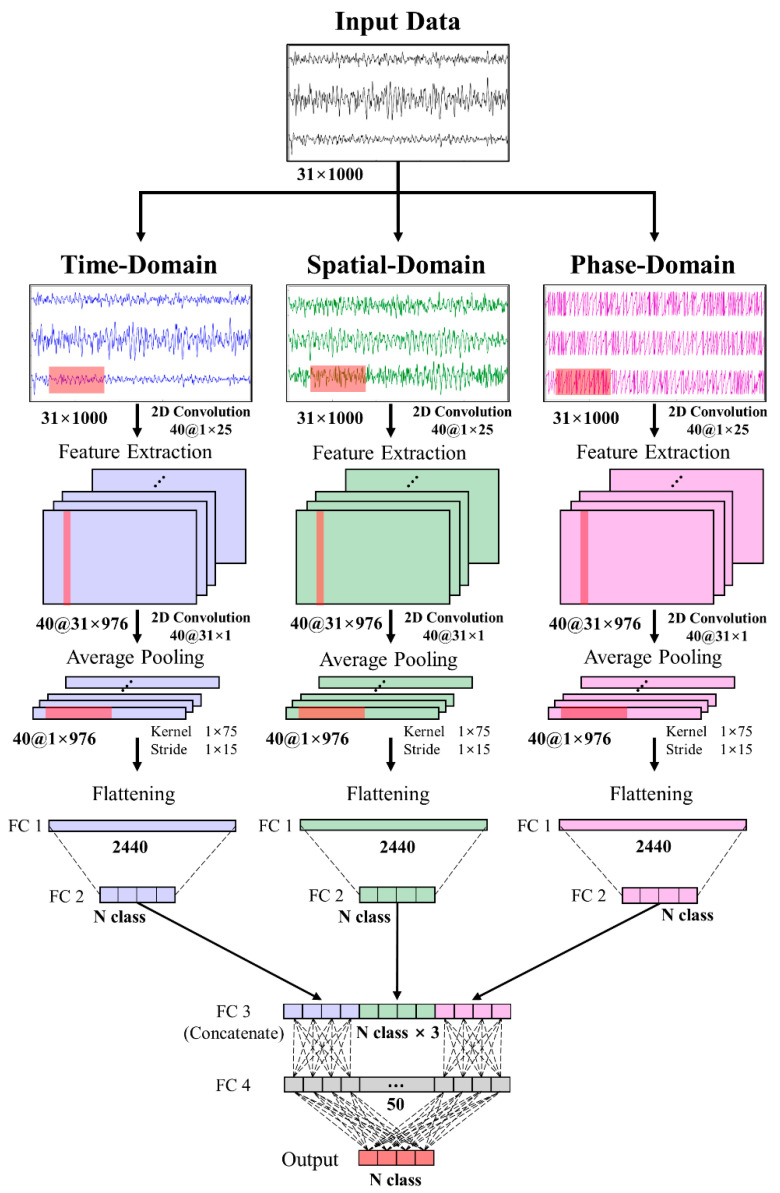
Block diagram of the MD-CNN model’s architecture. Input data are preprocessed into three domains, where each plane represents a feature map, and the features extracted from each multilayer are concatenated.

We propose an MD-CNN model to reduce the classification performance gap between dry and wet electrode BCIs by improving the classification performance of the model with a dry electrode BCI system. The proposed MD-CNN uses the architecture of a multilayer deep learning model for the time, spatial, and phase domains. From each CNN layer, time-domain features are extracted from the temporal features such as amplitude fluctuations [35], spatial-domain features are extracted from signals with maximized variance differences between different classes by spatial filters [41,42], and phase-domain features are extracted from the instantaneous phase [43]. The Fully Connected (FC) layer then combines the EEG features of the three domains and classifies them by weighting the features extracted from the most suitable domain for each subject or electrode type (Figure 1). First, the layers in each domain adopt the architecture of the ShallowConvNet model [35] to extract features from the input data preprocessed into the three domains, and the neural network layer (FC layer) combines the features of each domain and classifies the MI BCI. The multiple outputs are then used to train the model, to check the training results for each domain, and to obtain and combine the trained domain-specific parameters.

The MI classifier model was trained and evaluated with a randomly selected training set and test set using 10-fold cross-validation. The data were selected by applying a stratified method that maintained the class ratio so that the percentage of samples for each class was preserved. Therefore, for example, in a dataset with a total of 90 trials, 81 trials (27 trials × 3 classes) per fold were used for the training data, and the remaining 9 trials were used for the test data. To augment the data, the sliding window procedure [35,44] was performed by sliding a 4-s window at 0.1-s intervals along the data and cropping them. As a result of the sliding window data augmentation, a training set of 81 × 11 and a test set of 9 × 11 were created.

The input data were each domain with a fixed size of 31 × 1000 (channels × samples). The data were extracted through the first CNN layer with a receptive field size of 1 × 25 [35,45] and the second CNN layer with a receptive field size of 31 × 1. The first CNN layer had 40 receptive fields and the second had 40 receptive fields. Since each receptive field had a different value, the size of the extracted feature map that passed the previous two CNN layers was 40 × 1 × 976. Next, through a batch normalization layer, interlayer recentering and rescaling were performed and activated with a square function, and the data were compressed to 40 × 1 × 44 by an average pooling layer with a 1 × 75 kernel and a 1 × 15 stride. Activation and dropout were performed sequentially with a log function at a rate of 0.5 to train the EEG features. FC layers were used for weight learning and output feature generation for concatenation in each domain. In order to reduce overfitting, we used the maxnorm weight constraint [46,47], which imposes constraints on the weight vectors for all neurons in the CNN and FC layers in addition to the sliding window data augmentation. Finally, the output features of the time-, spatial-, and phase-domain representations were combined through the concatenated layers and again through the FC layer to perform weight learning for the final output. Activation of the FC layers for the final output was performed with the softmax function.

To learn and combine the output features for each domain layer, we performed weight learning for the class label output in each domain. The epoch of model learning was 100 and the batch size was 32. The loss was calculated using cross-entropy, and the Adam optimizer was adopted for model learning with a reduced learning rate, which was adjusted according to the loss reduction. In addition, the model weights and hyperparameters that produced the lowest validation loss with the test data were used to generate the predictive results.

### 2.2. Multi-Domain Input Preparation

#### 2.2.1. Spatial-Domain Representation with a Common Spatial Pattern

Spatial-domain representation was implemented through the common spatial pattern (CSP), which uses a spatial filter to identify the internal space of the signal in which the variance difference is maximized [41]. Multiclass CSP can be performed by implementing a binary-class CSP for each combination of classes or by using the joint approximate diagonalization (JAD) algorithm [42]. In our study, we implemented multiclass CSP with JAD because we wanted to use the spatially filtered signal as input data.

The EEG signal *E* of class *M* has the input format N × T (channel N). In the response, CSP must obtain a covariance matrix *W* to maximize the variance difference to obtain the spatially filtered signal *Z*. The projection matrix *W* is represented by *R*, indicating the representation of EEG data, and *D*, indicating the diagonal matrices in the following covariance matrix expression, which depends on the class condition:

(1) Z=WΕ

(2) $$W^{T}R_{X|c_{i}}W=D_{C}{}_{i},\;i=1,\;2,\;\ldots ,\;M$$

The JAD algorithm allows us to obtain the multiclass transformation projection matrix *W* by selecting the *L* columns of *W* that maximize the expression of mutual information.

(3) $$\begin{array}{cc} I\left(c,\;w_{j}^{T}x\right)\approx & -\sum_{i=1}^{M}P\left(c_{i}\right)\log\sqrt{w_{j}^{T}R_{x|c_{i}}w_{j}}\\ & -\frac{3}{16}{\left(\sum_{i=1}^{M}P\left(c_{i}\right)\left({\left(w_{j}^{T}R_{x|c_{i}}w_{j}\right)}^{2}-1\right)\right)}^{2} \end{array}$$

#### 2.2.2. Phase-Domain Representation with the Hilbert transform

Phase-domain representation is implemented through the Hilbert transform [43], which quantifies increases and decreases in the EEG data to calculate the instantaneous amplitude and phase (ϕ). The instantaneous phase of the analytical signal z(t) is obtained by the following expression:

(4) $$\begin{array}{c} z\left(t\right)=s\left(t\right)+j\hat{s}\left(t\right)=A\left(t\right)e^{j\phi \left(t\right)}\\ \hat{s}\left(t\right)=\frac{1}{\pi }\int_{-\infty }^{\infty }\frac{s\left(\tau \right)}{t-\tau }d\tau \end{array}$$

(5) $$\hat{s}\left(k\right)=\{\begin{array}{c} \frac{2}{\pi }\underset{n\;odd}{\sum }\frac{f\left(n\right)}{k-n};k\;even\\ \frac{2}{\pi }\underset{n\;even}{\sum }\frac{f\left(n\right)}{k-n};k\;odd \end{array}\;,\;n=-\infty ,\ldots ,-1,0,1,\ldots ,\infty$$

(6) $$\phi \left(k\right)=\arctan\left(\hat{s}\left(k\right)/s\left(k\right)\right)$$

where $\hat{s}$(t) is the Hilbert transform of *s*(*t*), a single channel’s time-domain data. With $\hat{s}$(k) of discrete Hilbert transformation [48], ϕ(k) is the instantaneous phase data of the signal, which was defined as the phase-domain representation of the signal and used as input to one of the multilayers of the model.

### 2.3. Public Dataset

To evaluate the proposed MD-CNN, we used the BCI Competition IV dataset 2a, consisting of EEG data for 4 MI classes (left hand, right hand, both foot, and tongue) collected from 9 healthy subjects over training and evaluation sessions. It was sampled at 250 Hz by 22 Ag/AgCl wet electrodes, with 72 trials per class and a total of 288 trials in each session. The MI EEG data were the MI period of 4 s after the cue and were lowpass filtered at 38 Hz [35] using a fourth-order zero-phase Butterworth infinite impulse response (IIR) filter. The steps for the input representation described above were then preprocessed by performing CSP (spatial-domain) and Hilbert transform (phase-domain) to generate the input dataset for the MD-CNN model. Finally, normalization was performed for each domain of the preprocessed data to scale before training the classifier.

### 2.4. Experimental Dataset

#### 2.4.1. Subjects

Ten healthy subjects (5 males and 5 females, all right-handed and 23–44 years of age) participated in 2 experiment sessions each. Eight out of 10 subjects had no previous experience of participating in BCI experiments, and none had a history of central nervous system abnormalities or related medical histories. Prior to the experiment, the subjects were informed about the experimental protocols, which were approved by the Institutional Review Board of Korea Institute of Science and Technology (KIST IRB number 2020-025; date of approval: 29 October 2020) and were conducted according to the guidelines of the Declaration of Helsinki, and they provided consent to participate in the study.

#### 2.4.2. Experimental Setup

The MI task performed in our experiment included two imagery tasks related to lower limb movement (gait and sitting down) and a resting state. During the MI task, the subject stood on crutches in front of a monitor adjusted to eye level and performed a mental rehearsal [15,49]. When the subjects were ready to perform the trial, they were instructed to start by pressing the button attached to the crutch. After the button had been pressed, the monitor showed a 3-s fixation cross with a beep and then presented a 2-s random cue, an upward arrow, a box, or a downward arrow, representing walking, resting, and sitting, respectively; the subject was then asked to perform the corresponding MI task for 5 s. After the 5 s of the MI task, a second beep sound played, notifying the subject of the end of the task and telling them to be ready for the next trial.

**Figure 2:**
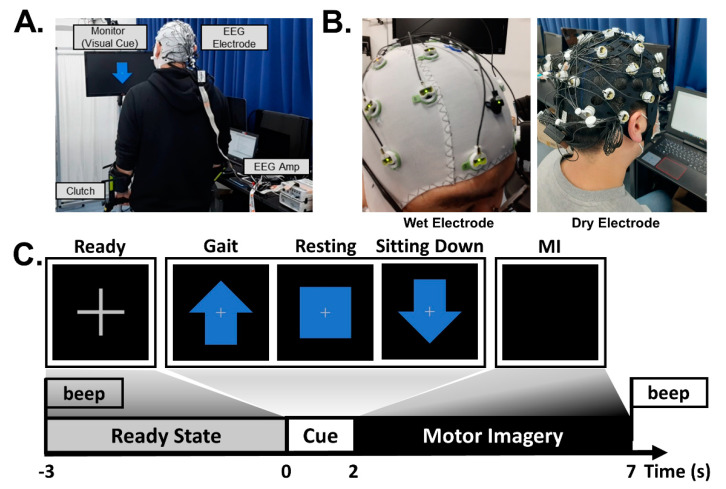
Schematic overview of the experimental protocol and setup. (**A**) Experimental environments and setup. (**B**) The two types of electrodes used in this study. (**C**) Experimental protocol of the two sessions: dry and wet electrode BCI.

The experiment consisted of 2 sessions (1 for dry electrode BCI and the other for wet electrode BCI) presented 1 hour apart on the same day. The session order was randomized, and the subjects were instructed to keep the MI used between the 2 sessions as similar as possible. Each session consisted of 30 trials per class, with a total of 90 trials recorded per session, during which the subjects were asked to minimize blinking and body movement during the 5-s MI task, especially for MI related to body sensations and motor execution. Figure 2 shows a schematic overview of the experiment, including the two types of electrode devices used in this study, the experimental environment and setup, and an example of the experimental protocol.

#### 2.4.3. Data Acquisition

The EEG data were measured using both wet electrodes (actiCAP Slim, Brain Product GmbH, Gilching, Germany) and dry electrodes (actiCap Xpress Twist, Brain Product GmbH, Gilching, Germany) and a BrainAmp device (actiCHamp, Brain Product GmbH, Gilching, Germany) by selecting 31 channels from the international 10-20 system (FP1, FP2, F3, F4, F7, F8, FC1, FC2, FC5, FC6, C1, C2, C3, C4, Cz, CP1, CP2, CP5, CP6, P3, P4, P7, P8, Pz, TP9, TP10, O1, O2, Oz, PO9, and PO10). The reference and ground electrodes were placed at FCz and AFz, respectively. The impedance level was set to remain below 20 kΩ for the wet electrode system and 500 kΩ for the dry electrode system during the experiment. The sampling rate was 500 Hz, and a 60 Hz notch filter was applied to remove power line noise.

#### 2.4.4. Signal Processing

The EEG data were bandpass filtered from 4 to 40 Hz [50,51,52] using a fourth-order zero-phase Butterworth infinite impulse response (IIR) filter [15] and subsequently downsampled to 250 Hz during preprocessing to avoid overfitting and reduce the number of deep learning parameters required. We then performed preprocessing and normalization for input representation before training the classifier, as with the public dataset above.

## 3. Results

### 3.1. MD-CNN’s Classification Accuracy in Public Dataset

**Figure 3:**
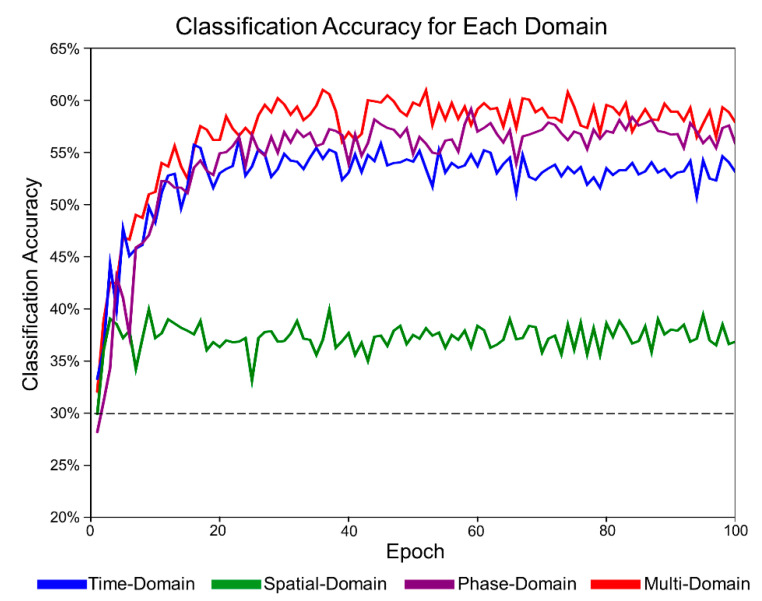
Classification accuracy of the MD-CNN in each domain for S6 with the BCI Competition IV dataset 2a: time-domain (blue), spatial-domain (green), phase-domain (purple), and multi-domain representation (red). The horizontal dotted line indicates the chance level.

**Figure 4:**
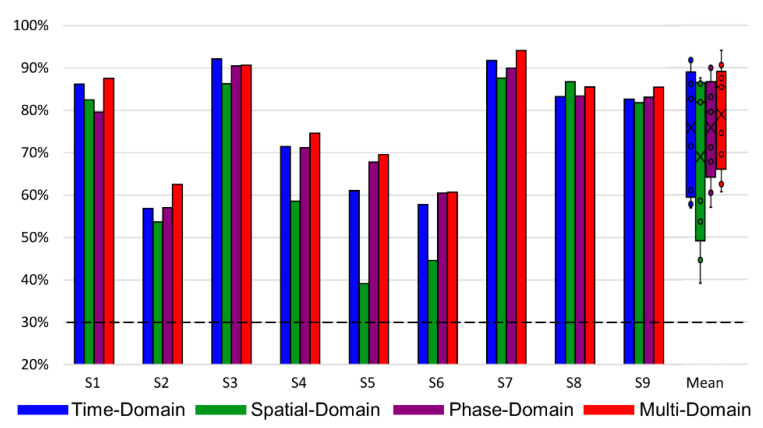
Classification accuracy of the MD-CNN for each domain and subject in the BCI Competition IV dataset 2a: time-domain (blue), spatial-domain (green), phase-domain (purple), and multi-domain representation (red). The box plots with scatter points depict the mean value and distribution for each domain. The horizontal dotted line indicates the chance level.

We compared the classification accuracy of the proposed MD-CNN as a four-class classifier with conventional models (FBCSP [53], EEGNet [54], ShallowConvNet [35], and DeepConvNet [35]) for the BCI Competition IV dataset 2a. The chance level considering Müller-Putz et al.’s confidence limit was about 30% [55]. As shown in Table 1, the MD-CNN model had the highest classification accuracy (78.96%) among the comparative models (FBCSP: 67.90%; EEGNet: 67.32%; ShallowConvNet: 72.73%; DeepConvNet: 67.25%) (one-way ANOVA; *p* = 0.375). In most subject-specific classification results, MD-CNN was the highest among the comparative models; in particular, S6 in Figure 3, which showed the largest performance improvement over the other models, was further investigated for multi-domain and single-domain classification results. Of the multi-domain classification accuracy (60.71%) and the single-domain classification accuracy (time: 56.31%; spatial: 39.03%; phase: 59.14%) in S6, the multi-domain classification accuracy was the highest. Although there was no statistically significant difference between the multi-domain and the other domains (time, spatial, phase) (one-way ANOVA; *p* = 0.554), the multi-domain showed the highest classification accuracy compared with domain-specific results, except for S3 and S8 (Figure 4).

### 3.2. MD-CNN’s Classification Accuracy on the Experimental Dataset

#### 3.2.1. MD-CNN Model Evaluation in Dry–Wet Electrode BCI Experiments

**Figure 5:**
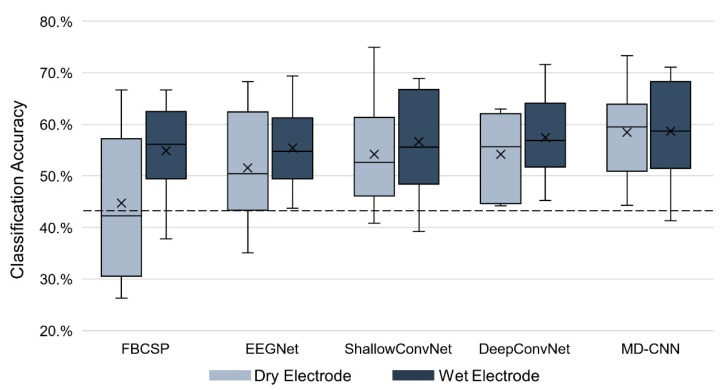
Classification accuracy of the FBCSP, EEGNet, ShallowConvNet, DeepConvNet, and MD-CNN models. The horizontal dotted line indicates the chance level.

We compared the classification accuracy of the proposed MD-CNN model as a three-class classifier with that of other classifiers (FBCSP, EEGNet, ShallowConvNet, and DeepConvNet) for dry and wet electrodes. The chance level considering the confidence limit was about 43.33% [55]. Figure 5 and Table 2 show the classification accuracy for several MI BCI models and electrode types. The MD-CNN model had the highest average classification accuracy for both electrode types among the compared models (58.44% for the dry-type BCI and 58.66% for the wet-type BCI). Moreover, our results showed that deep learning-based classifiers outperformed the FBCSP classifier in dry (one-way ANOVA; *p* = 0.0778) and wet (one-way ANOVA; *p* = 0.891) electrode BCI. Furthermore, the difference in accuracy between the machine learning-based classifier (FBCSP: 44.74%) and the deep learning-based classifiers (EEGNet: 50.61%; ShallowConvNet: 54.17%; DeepConvNet: 54.20%; MD-CNN: 58.44%) using dry electrodes was larger than that using wet electrodes.

Table 2 shows that the differences in classification accuracy according to electrode type for the MI BCI models were 10.15% for FBCSP, 3.87% for EEGNet, 2.49% for ShallowConvNet, 3.26% for DeepConvNet, and 0.22% for MD-CNN. Although there was no statistically significant difference in the classification accuracy between the dry and wet electrodes for any classifier (paired *t*-test, *p* > 0.05), the deep learning-based classifiers tended to have smaller differences in accuracy between the dry and wet BCI systems than the machine learning-based classifiers by having better accuracies for the dry electrode BCI. The effect size of each classifier, calculated as Cohen’s d, was 0.5816 for FBCSP, 0.4474 for EEGNet, 0.1994 for ShallowConvNet, 0.6044 for DeepConvNet, and 0.0292 for MD-CNN.

#### 3.2.2. MD-CNN’s Domain-Specific Classification Accuracy

**Figure 6:**
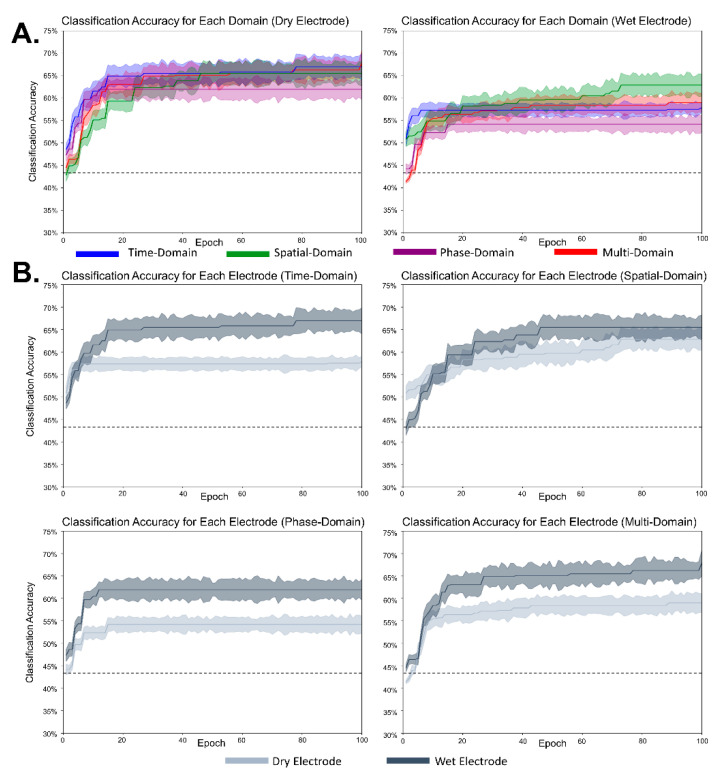
Classification accuracy of the MD-CNN model for S10. The shading represents the standard deviation according to cross-validation. The horizontal dotted line indicates the chance level. (**A**) Classification accuracy for each domain per electrode: time-domain (blue), spatial-domain (green), phase-domain (purple), and multi-domain representation (red). (**B**) Classification accuracy for each electrode per domain: dry electrode (light blue) and wet electrode (dark blue).

Figure 6 shows the classification accuracy for each domain for the MD-CNN model to confirm that each of the domain-specific learning results was dependent on the electrode type. We investigated the MD-CNN’s classification accuracy for time-domain (blue), spatial-domain (green), phase-domain (purple), and multi-domain representations (red), which were produced by combining and learning the features of the three individual domains.

**Figure 7:**
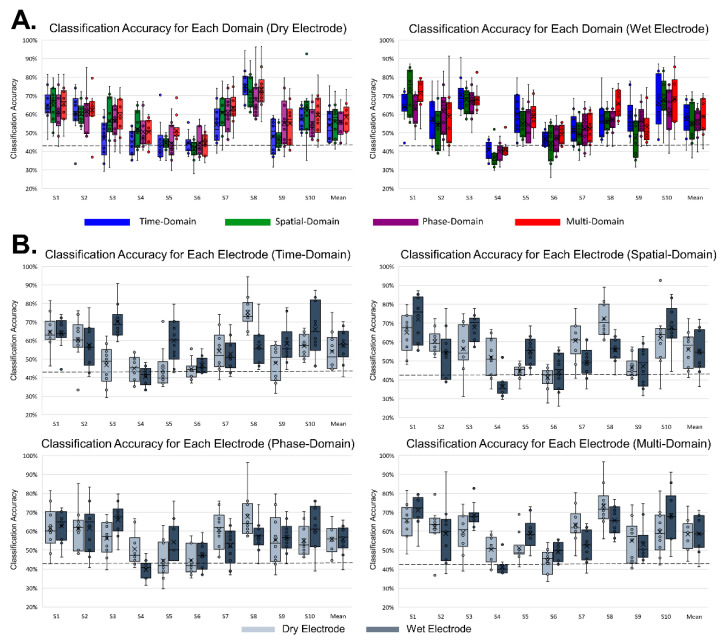
Classification accuracy of MD-CNN for all subjects. The boxplots with scatter points are drawn from 10-fold cross-validation results for each subject. The horizontal dotted line indicates the chance level. (**A**) Classification accuracy for each domain per electrode: time-domain (blue), spatial-domain (green), phase-domain (purple), and multi-domain representation (red). (**B**) Classification accuracy for each electrode for each domain: dry electrode (light blue); wet electrode (dark blue).

Figure 7 shows the classification accuracy of the MD-CNN model separated by domain for each subject or electrode type. For wet electrode BCI (Figure 7B), the classification accuracy of the time-domain representation was higher than that of the other two domains (time domain: 57.33%; spatial domain: 54.98%; phase domain: 55.83%), but this did not hold for the dry electrode BCI (Figure 7A) (time domain: 54.06%; spatial domain: 56.05%; phase domain: 55.72%). The multi-domain classification accuracy either followed the classification performance of the highest performing domain for each subject or was the highest of all classification performances. Although there was no statistically significant difference (one-way ANOVA; dry: *p* = 0.762; wet: *p* = 0.807) between the multi-domain and the other domains (time, spatial, phase), multi-domain classification showed the highest performance in dry and wet electrode MI BCI systems.

## 4. Discussion

In this study, we proposed an MD-CNN model with a multilayer structure for three domains and investigated the possibility of dry electrode MI BCIs. Before training the proposed MD-CNN model on data collected in this study, we evaluated the proposed MD-CNN on the BCI Competition IV dataset 2a and identified the classification accuracy (78.96%). After that, 10 subjects performed a three-class lower-limb MI BCI over two sessions with dry and wet electrodes. We also compared the classification accuracy of the proposed MD-CNN model with that of other classifiers. We demonstrated that the MD-CNN model showed a higher classification accuracy for both the dry (58.44) and wet (58.66) BCI systems and a smaller difference in accuracy between the two than the other existing classifiers.

### 4.1. Classification Performance of MD-CNN with the Public Dataset

Some BCI studies [35,54] have sought to improve the classification accuracy of MI BCIs through a deep learning approach. ShallowConvNet [35], which learns spatial information and time information from the raw EEG data, has frequently been used as a comparative model in this deep learning approach and reported 72.05% classification performance for four class MI tasks using the BCI Competition IV dataset 2a in a model reimplemented directly in this study. Recent studies [56,57] have also proposed a model that leverages information from other domains rather than just a single layer that extracts only spatial and time information. Sakhavi et al. [57] proposed CW-CNN that generates and learns the input structures with spatial and time information, and they reported a classification accuracy of 74.46% for four-class MI task classification using the BCI Competition IV dataset 2a. Likewise, Amin et al. [56] proposed an MCNN model that allows multiple features to be extracted through a multilayer network of different depths. They reported a classification accuracy of 75.72% for four-class MI tasks using the BCI Competition IV dataset 2a and compared it with other models.

In this study, we aimed to investigate whether a deep learning model that learns multi-domain input data in parallel with multilayer structures could combine and utilize the features of the multi-domain to improve the classification performance of dry electrode BCIs. The proposed MD-CNN learned multi-domain input data from three domains via three parallel layers, demonstrating a high classification accuracy of 78.96% for four-class MI tasks using the BCI Competition IV dataset 2a (Table 1). The reason why MD-CNN outperformed other models can be inferred from the classification accuracy for each domain of S6 (Figure 3), one of the most improved subjects. The classification accuracy in the time domain, which was mainly used in other models, was similar to other models. The augmentation of the phase domain may have helped MD-CNN improve its classification results. Together with Figure 4, these results suggest that MD-CNN outperformed the existing single-domain-based algorithms by learning the features from multiple domains in parallel and weighting the features of domains appropriate for the specific subject, as intended by the design.

### 4.2. Classification Performance of MD-CNN with Dry and Wet Electrode MI BCIs

After confirming its applicability through MD-CNN’s high performance in MI task classification with the public dataset, our study aimed to achieve its original purpose of reducing the difference in MI BCI classification accuracy between dry and wet electrode systems by proposing a novel model appropriate for dry electrode BCIs. Figure 6 and Figure 7 show that the classification results of each domain’s features varied by subject and electrode type. Among the domain-specific classification accuracies for S10, the spatial-domain accuracy was the highest (66.11%) with the dry electrode system, while the time-domain accuracy (67.96%) was the highest with the wet electrode system. This indicates that the MI-related EEG features have different domain-related characteristics depending on the electrode type, even for the same subject. Furthermore, the existence of subject-specific domain characteristics, as shown in Figure 7, is congruent with other studies demonstrating that different subjects had different best CNN parameters [36,56] or inputs [36,37,38]. Across the entire subject cohort, the time-domain representation with the wet electrode BCI showed the highest average classification accuracy (57.52%) after multi-domain BCI with 58.66%, but it had the lowest accuracy (54.06%) of the three domains with dry electrode BCI. These results show that MD-CNN improves classification accuracy by extracting and learning the EEG features that are less affected by the low SNR of dry electrodes across multiple domains, as intended.

Four classifiers (FBCSP, EEGNet, ShallowConvNet, and DeepConvNet) were evaluated using the same conditions as MD-CNN to compare existing dry and wet electrode BCI classifiers. To the best of our knowledge, we are the first to compare the MI BCI classification accuracy for the same subjects with both dry and wet electrodes using CNN-based classifiers. As shown in Figure 5, the accuracy of the CNN-based classifiers was higher for both dry and wet electrode BCIs than FBCSP, and the difference in the accuracies between the two BCI systems was smaller than that with FBCSP. This might be associated with the fact that CNN-based classifiers were shown to be robust to perturbation-induced noise [35,58]. The noise caused by the low SNR of the dry electrode BCI system cannot be explained only by perturbations or random noises. Along with the results that the deep learning-based classifiers, including the proposed MD-CNN, showed a higher tendency than the machine learning-based FBCSP classifier in a dry electrode MI BCI (one-way ANOVA; *p* = 0.0778), the proposed MD-CNN model showed the possibility that deep learning-based classifiers could improve the classification performance of dry electrode system-based MI BCIs. Consequently, we also confirmed that the smallest difference in classification accuracy (0.22%, *p*-value = 0.93, effect size = 0.0292) between the wet and dry electrode MI BCIs was achieved with the proposed MD-CNN.

### 4.3. Limitations and Future Work

The main limitation of this study was that the proposed MD-CNN model showed an improvement against other algorithms for the public dataset and the dry electrode MI BCI experiment dataset but in the wet electrode MI BCI experiment dataset. These limitations might be related to the characteristics of MD-CNN, which exploits an approach in which each subject or each electrode has a specific domain with appropriate features for MI BCI classification. MD-CNN’s specific domain classification results for dry electrode MI BCI or the public dataset had higher results for domains other than the time domains, which are frequently used in existing algorithms, but MD-CNN’s specific domain classification results for the wet electrode MI BCI with little improvement had the highest results from the time domains (Figure 4 and Figure 7). We conjecture that a wet electrode BCI with proper time-domain features within the multi-domain would have no significantly improved classification accuracy compared with existing algorithms that utilize the same proper time-domain features. As already noted, only a few studies have compared MI with dry and wet electrode BCIs, and none have compared classification accuracies with those of CNN-based classifiers. Moreover, no studies have used three classes of lower-limb MI in a BCI, and thus we were unable to compare our accuracy with that of other studies. It is warranted to further improve classification accuracy by adopting different network structures for different domains to accommodate more appropriate features for each domain. Nevertheless, it is difficult to overcome the criticism that the MI BCI classification accuracies of dry and wet electrodes have become similar due to their low baseline of classification accuracy compared with other studies [56,59,60] related to other motor imagery tasks. Therefore, further research should collect and analyze the wet and dry electrode BCI data using more common motor imagery (e.g., right and left hands) that can be compared with the accuracy of other studies [37,40].

The main goal of reducing the difference in MI BCI classification accuracy between dry and wet electrode BCIs can be supported by the low effect size (d = 0.0292) with the proposed MD-CNN [61]. The statistically significant improvement in the performance of dry and wet electrode BCIs using the proposed MD-CNN over other models was not a precondition for the goal of reducing the performance gap between dry and wet electrode BCIs [62]. However, the proposed MD-CNN did not show a statistically significant improvement over other models in dry and wet electrode BCIs due to the small number of subjects [63,64]. Therefore, in future studies, sufficient subjects should be secured to confirm that the proposed MD-CNN has better classification performance over other models in dry and wet electrode BCIs. Identifying differences in the classification accuracy of the electrodes with CNN algorithms has been considered an important unexplored issue to date, and this has been achieved through this study.

## 5. Conclusions

In this study, we investigated the MI BCI classification performance with dry and wet electrodes by using CNN-based algorithms. We also proposed MD-CNN, a model based on a multi-domain CNN model with a multilayer architecture, and confirmed its classification performance with the BCI Competition IV dataset 2a. The classification performance of MD-CNN in the public dataset showed an improved accuracy of 78.96% over other algorithms and appropriate subject-specific domain characteristics. The comparative analysis with the experimental dataset showed that the difference in performance between the two electrode systems was reduced with CNN-based algorithms and was the smallest with MD-CNN. Furthermore, the proposed MD-CNN improved the classification performance by combining domains with EEG features suitable for MI classification, which may vary by subject or electrode type. These experimental results showed the possibility of using dry electrodes in the MI BCI field by developing classification algorithms based on deep learning.

## Figures and Tables

**Table 1:** Classification accuracy of FBCSP, EEGNet, ShallowConvNet, DeepConvNet, and MD-CNN for the BCI Competition IV dataset 2a.

Subject	FBCSP	EEGNet	Shallow ConvNet	Deep ConvNet	MD-CNN
S1	78.47	76.10	84.61	77.43	87.56
S2	53.53	47.97	53.94	49.42	62.50
S3	83.80	91.61	90.74	87.15	90.63
S4	60.59	51.91	65.80	51.04	74.59
S5	60.19	56.77	53.82	60.24	69.56
S6	47.74	51.04	52.03	51.74	60.71
S7	90.57	71.12	88.66	74.94	94.10
S8	70.49	77.84	82.81	76.04	85.53
S9	65.74	81.54	82.18	77.26	85.47
mean (s.d.)	67.90 (14.21)	67.32 (15.74)	72.73 (16.19)	67.25 (14.17)	78.96 (12.43)

**Table 2:** Comparison of classification accuracy between dry and wet electrode BCI systems.

Classifier	Dry Mean (s.d.)	Wet Mean (s.d.)	*p*-Value	Effect Size Cohen’s d
FBCSP	44.74 (14.26)	54.89 (9.37)	0.10	0.5816
EEGNet	51.57 (10.70)	55.44 (8.03)	0.22	0.4132
ShallowConvNet	54.17 (10.66)	56.66 (10.13)	0.54	0.1940
DeepConvNet	54.20 (7.85)	57.46 (8.22)	0.09	0.6044
MD-CNN	58.44 (9.76)	58.66 (9.76)	0.93	0.0292
